# Coordinate Dependence of Variability Analysis

**DOI:** 10.1371/journal.pcbi.1000751

**Published:** 2010-04-22

**Authors:** Dagmar Sternad, Se-Woong Park, Hermann Müller, Neville Hogan

**Affiliations:** 1 Department of Biology, Northeastern University, Boston, Massachusetts, United States of America; 2 Department of Electrical & Computer Science, Northeastern University, Boston, Massachusetts, United States of America; 3 Department of Physics, Northeastern University, Boston, Massachusetts, United States of America; 4 Department of Movement Science, Justus-Liebig University, Giessen, Germany; 5 Department of Mechanical Engineering, Massachusetts Institute of Technology, Cambridge, Massachusetts, United States of America; 6 Department of Brain and Cognitive Sciences, Massachusetts Institute of Technology, Cambridge, Massachusetts, United States of America; University College London, United Kingdom

## Abstract

Analysis of motor performance variability in tasks with redundancy affords insight about synergies underlying central nervous system (CNS) control. Preferential distribution of variability in ways that minimally affect task performance suggests sophisticated neural control. Unfortunately, in the analysis of variability the choice of coordinates used to represent multi-dimensional data may profoundly affect analysis, introducing an arbitrariness which compromises its conclusions. This paper assesses the influence of coordinates. Methods based on analyzing a covariance matrix are fundamentally dependent on an investigator's choices. Two reasons are identified: using *anisotropy* of a covariance matrix as evidence of preferential distribution of variability; and using *orthogonality* to quantify relevance of variability to task performance. Both are exquisitely sensitive to coordinates. Unless coordinates are known *a priori*, these methods do not support unambiguous inferences about CNS control. An alternative method uses a two-level approach where variability in task execution (expressed in one coordinate frame) is mapped by a function to its result (expressed in another coordinate frame). An analysis of variability in execution using this function to quantify performance at the level of results offers substantially less sensitivity to coordinates than analysis of a covariance matrix of execution variables. This is an initial step towards developing coordinate-invariant analysis methods for movement neuroscience.

## Author Summary

Over the past decade the identification of synergies has become a prominent theme in motor neuroscience. Like other aspects of neural organization (e.g., vision) the control of coordinated movement is almost certainly hierarchical with synergies a key feature of this hypothesis. In pursuit of identifying synergies, whether flexible or hard-wired in biomechanical or physiological structures, many studies have analyzed variability with techniques of dimensionality reduction such as principal component analysis. Results have been interpreted as evidence for controlled variables in motor control. Our paper demonstrates that such analyses and conclusions based on these methods are exquisitely sensitive to the coordinates of the variables that are the basis for this analysis. As these coordinates are often chosen for convenience of measurement or analysis, any conclusions about neural control are therefore ambiguous at best. The development of coordinate-independent analyses was an important step in the development of modern physics. Here we highlight the problems induced by coordinate-dependency in studies of neural control and present initial steps towards coordinate-independent analyses relevant to computational biology. We critically examine an alternative method proposed to analyze variability for identification of structure and show that it is significantly less sensitive to assumed coordinates than conventional analyses.

## Introduction

A study of multivariable behavior naturally raises the question of which reference frames the central nervous system (CNS) may use to coordinate its actions. For example, Morasso [1] studied planar reaching movements and showed that translation and rotation of the start and target positions evoked *systematic variation* of joint kinematics (angles of shoulder and elbow) but much less variation of hand kinematics (Cartesian coordinates of the hand). This indicated that hand motion in “visual space” is an important consideration in central coordination of these movements. That implied a need for the CNS to transform between representations in different coordinates, e.g. visual to motor, as one challenge of coordination and control. Evidence that at least one such transformation is implemented in the parietal cortex was presented by Andersen and Zipser [2]. Soechting and Flanders [3] provide a comprehensive review of other evidence from eye, head, and body movements elicited by vestibular and visual stimuli and arm movements with their neural correlates in motor cortex.

*Stochastic variation* provides another source of evidence about how the CNS may control and coordinate behavior. Patterns in variability—especially when they are invariant across experimental conditions—can reveal underlying control strategies that are inaccessible to direct measurement. The structure of variability over repeated performances can be especially meaningful when a task is redundant, i.e., the task presents a multiplicity of equivalent ways to achieve the same end goal. A paradigmatic example is multi-joint movement, where the limbs have more degrees of freedom than minimally required to perform an intended task. Structure in this variability can reveal the organization of the neuromechanical control system. In a study of the postural responses of cats to tilting of their support surface, Lacquaniti and Maioli [4], [5] showed that while three joint angles of the limbs (scapula, shoulder and elbow of the forepaw; hip, knee and ankle of the hindpaw) exhibited a large variability (on the order of 30°) they co-varied to lie close to a plane within the three-dimensional configuration space. This is presented as evidence of a *synergy* that reduces the dimensionality of the control problem—one solution to the “degrees-of-freedom” problem [6].

An obvious but critical fact is that the structure of *observed variability* is defined in a space with coordinates selected by the researcher. There is no a priori reason to believe that these *external* coordinates are the same as any *internal* coordinates of a putative neural representation. For example, in the analysis of multi-joint limb movements, these coordinates may be the angles of the biomechanically defined joints. However, joint angles can be described following many different conventions as many standard textbooks in biomechanics and robotics document [7], [8]. While these alternative angle conventions provide equivalent descriptions of physical reality, the choice becomes important when the focus is on inferring CNS control strategies for multi-joint movement generation. In fact, the question of which coordinates or control variables may be represented in the CNS is a deep and difficult problem that lies at the heart of the study of motor control. Until that question is answered, the coordinates for data analysis remain a choice of the experimenter. If this choice should affect the outcome of the analysis, an uncomfortable arbitrariness would result.

In which coordinate space should patterns be sought? Does the structure of variability change when the problem is described in alternative coordinates? To what degree do the conclusions drawn from analyses in alternative coordinates agree? In this paper we show that some widely-used methods of analysis intended to illuminate CNS control are exquisitely sensitive to assumed coordinates and cannot provide unambiguous inferences about control. We further show that an alternative method which includes two levels of variables—those that characterize task execution and those that quantify its result, with a function or mapping relating the two—promises to be less sensitive.

## Methods

Using the example of a redundant reaching task we simulated performance variability in two different joint angle coordinates that have been used in the literature. In accordance with the most widely-used methods, analysis of variability was based on the data covariance matrix. Because these approaches proved to be exquisitely sensitive to coordinate choice, we first analyzed the influence of linear coordinate transformations on orthogonality, a core assumption of covariance-based analysis. We then used the special eigenstructure of a covariance matrix to analyze the influence of linear coordinate transformations on the anisotropy of a data distribution, another core assumption of covariance-based analysis.

An alternative method based on two sets of variables (one describing how a task is executed, the other describing the corresponding result) was analyzed by examining the geometric structure of the function relating these two levels. In particular, we studied the manifold defined by the extremal values of this function and the curvature of the function in the neighborhood of that manifold. We analyzed the sensitivity of both of these geometric features to general transformations of the coordinates chosen for the execution variables.

To ground the abstract analysis in realistic data, we computed the three different quantitative features of performance variability–Tolerance, Noise, and Covariation (TNC)–in exemplary data of one subject performing a throwing task. The task and the method of computing these measures are detailed in Cohen and Sternad (2009). To test the sensitivity of this TNC method to the coordinates chosen for the execution variables, we considered two plausible choices that are related by a non-trivial nonlinear transformation. We conducted the analysis in these two coordinate frames and compared the resulting variation observed in 2,880 performance attempts over a period of 16 days. To understand the outcome of this comparison we analyzed the sensitivity of these three measures of performance variability to linear and nonlinear transformations of the coordinates chosen for the execution variables.

## Results

### Covariance-Based Analysis

One reasonable approach to identifying structure in variability is to focus on the covariance matrix derived from a set of observations. This is at the heart of principal component analysis and also many other related methods. The difficulties are perhaps best illustrated by the so-called Un-Controlled Manifold (UCM) method which purports to identify features of CNS control based on analysis of variability [9], [10], [11], [12]. Using multi-joint reaching as an exemplary task, the problem is how *n* execution variables (e.g., seven upper-extremity degrees of freedom, assuming the shoulder is at a fixed location in space and that the hand and fingers may be treated as a single rigid body) are coordinated to achieve an *m*-dimensional result (e.g., location of the hand in external Euclidean space with three degrees of freedom). Given *n*>*m*, a multiplicity of solutions exist that equally satisfy the task requirement. Further, for every particular hand location, the set of solutions form a *manifold* in the space of execution variables, e.g., joint angles. This manifold may be visualized as analogous to a curved surface in execution space; every point on the surface corresponds to a combination of joint angles that yield the same hand location. Changes of the joint angles that are coordinated to remain on that surface do not change the hand location.

To seek evidence of CNS control strategies, the UCM method examines variability over repeated performances. To simplify analysis, a locally linear approximation to the manifold is defined. Specifically, the *result variable* (hand position in space) is mathematically defined as a function of the *execution variables* (joint angles). The Jacobian matrix of that function—a matrix of partial derivatives of each result variable (hand position coordinate) with respect to each execution variable (joint angle)—is defined. In general, the Jacobian matrix varies with limb configuration but it can be evaluated at any point to yield a matrix of constants. In UCM analysis, the Jacobian matrix is typically evaluated at the mean of the observed distribution of execution variables. Using standard methods of linear algebra the Jacobian matrix is analyzed to identify its *kernel* or *nullspace*. The nullspace may be visualized as analogous to a plane that is tangent to the curved manifold at the evaluation point. It defines the “do-not-matter” directions: small changes of the execution variables about that point which are coordinated in such a way as to remain within that plane do not matter because they produce negligible changes in the result [13], [14]. Conversely, all deviations in the *orthogonal complement* of this nullspace affect the result. The orthogonal complement may be visualized as analogous to directions perpendicular to the tangent plane described above.

Scholz and Schöner [10] hypothesized that if execution variability is smaller in those directions for which the result is more sensitive to deviations than in those directions for which deviations do not matter, control is indicated. To quantify the degree of control, execution variability is projected onto the nullspace (the do-not-matter directions) and onto its orthogonal complement. If the variability per degree of freedom in the do-not-matter directions is larger than in the orthogonal directions, this is taken as evidence of skill, as control is not exerted where it does not matter. Hence, the manifold (and its tangent, defined by the nullspace of the Jacobian matrix) are termed the uncontrolled manifold [15].

In the early papers an explicit goal was to identify variables that may be controlled by the CNS. Using a shooting task as an example, Scholz, Schöner and Latash (2000) hypothesized that rather than controlling all elements of the arm directly, a candidate controlled variable was the orientation of the pistol barrel, as it ultimately determines the accuracy of pointing. An alternative variable was the center of mass of the arm configuration. Using these quantities to define the hypothesized task, and treating joint angles as execution variables, joint angle variability at selected points along the limb trajectory was assessed with respect to its effect on these two alternative result variables. Relatively more variability in the do-not-matter directions was taken as support for the orientation of the pistol barrel as the more likely candidate for a controlled variable than the center of mass of the arm.

The idea that the CNS focuses its control effort on variables that matter while allowing inevitable variability to be distributed along do-not-matter directions has considerable conceptual appeal. The same feature can be generated by a stochastic optimal feedback control strategy [16], [17] which has been proposed as a theory of CNS control. Unfortunately, although these studies pursue an important question in a hypothesis-driven way, this analysis of a covariance matrix has major weaknesses as we detail below. Given the general appeal of the idea, we also attempt to identify a means to overcome these weaknesses.

### Dependence on Coordinate Choice

**Figure 1:**
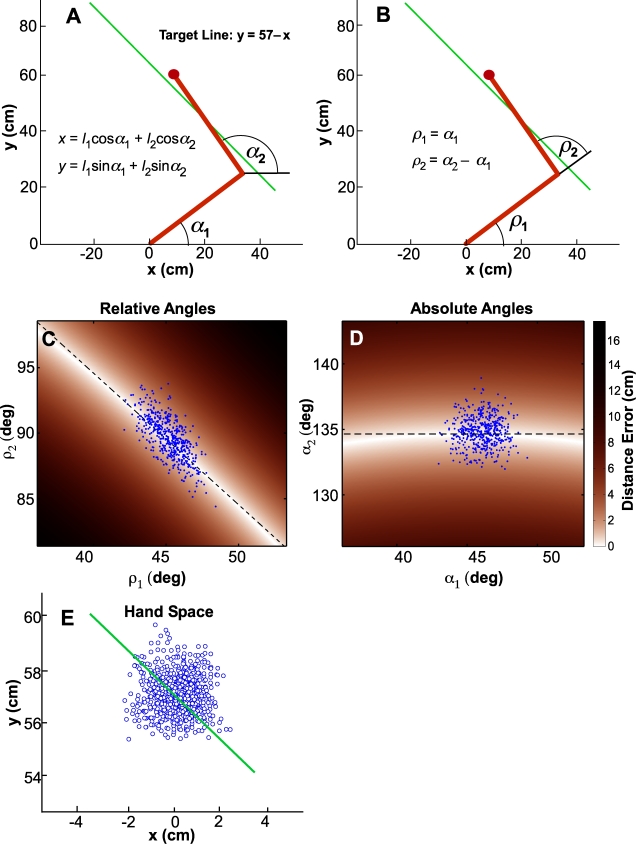
Sketch of a two-joint arm reaching to a target line as a simple example for a redundant task. The target line is defined in extrinsic coordinates, *x*, *y*. **A:** Illustration of absolute angle definitions of the shoulder and elbow joint, 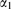 and 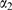, with respect to the shoulder axis. The relation between joint angles and extrinsic hand coordinates is given by the equations where *l*_1_ and *l*_2_ refer to the respective segment lengths, both 40 cm in the simulation. **B:** Relative joint angle definitions, 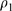 and 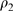. **C:** Simulated data displayed in the space of the relative joint angles, 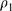 and 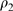. The color code denotes deviations from the target line, with darker colors referring to larger distances. The curved white line denotes the UCM, the set of solutions for which the end-effector is exactly on the target line. The dashed straight line denotes the nullspace of the Jacobian matrix evaluated at the mean of the data distribution, providing a linear approximation that is tangent to the UCM at that point. The data are aligned with the direction of the nullspace, i.e. show structure. **D:** The same simulated data displayed in the space of the absolute joint angles, 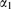 and 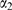. The same data are now *not* aligned with the direction of the nullspace, i.e. *do not* show structure. **E:** The same set of data displayed in extrinsic hand space, in which it was generated to have an isotropic random distribution with its mean centered on the target line.

To illustrate the general problem, consider a simplified hypothetical pointing task: reaching in the horizontal plane to point to a line. Assume the thorax is stationary and only the shoulder and elbow joints may move, so that the upper extremity may be modeled with only two segments. Assume the line is oriented diagonally with respect to a line through the shoulders (Figure 1A). Successful pointing is achieved by moving the hand to any location along the line. Because placing the hand at every location on a line achieves the same zero error, the task may successfully be completed with infinitely many combinations of joint angles.

Next, consider how the joint angles may be defined: two common conventions found in the literature are illustrated in the figure: “absolute” coordinates measured with respect to a stationary frame; and “relative” coordinates measured with respect to adjacent limb segments. Figure 1A illustrates absolute joint angles; the orientation of the upper arm, 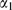, and forearm, 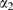, are both measured with respect to the same stationary reference, a line through the shoulders. Alternatively, Figure 1B illustrates relative joint angles; the orientation of the upper arm, 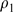, is measured as before but the orientation of the forearm, 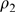 is measured with respect to a movable reference, the orientation of the upper arm. These are only two of an uncountably infinite set of alternatives, any of which fully define the configuration of the upper extremity. However, these two alternatives are related by simple linear equations: 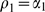 and 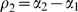 (see Figure 1B).

Absolute angles are advantageous because the forward kinematic equations expressing hand location in the horizontal plane as a function of limb configuration have a particularly compact form which simplifies computation of the Jacobian matrix (Scholz & Schöner, 1999). Note, however, that among the infinity of alternatives, there is no principled reason aside from computational convenience for giving primacy to either of these two conventions.

Assume a hypothetical set of 500 trials that scatter the hand location on and around the target line. If this set of data is represented in the space of relative angle coordinates, they exhibit the anisotropic distribution visible in Figure 1C. The color code denotes deviations from the target line, with darker colors referring to larger distances. The curved white line denotes the UCM, the set of joint angle combinations for which the endpoint is exactly on the target line and the deviation is zero. The dashed straight line denotes the nullspace of the Jacobian matrix evaluated at the mean of the data distribution, providing a linear approximation that is tangent to the UCM at that point. According to the rationale of the UCM method, this data distribution has structure such that the projection onto the UCM or its linearization is larger than the projection onto its orthogonal complement. The putative interpretation would be that this performance shows an ability to identify and take advantage of the redundancy of the task; the variability is not randomly scattered but channeled preferentially along the do-not-matter direction.

This interpretation would be premature. Consider Figure 1D, which shows exactly the same 500 data points but represented in the space of absolute joint angles: The data distribution which was previously anisotropic and well-aligned with the UCM becomes isotropic simply due to this change of coordinates. According to the logic of the UCM method, the putative interpretation would now be that the data shows no signs of this particular skill. Clearly, both interpretations cannot be supported simultaneously. In the absence of an objective argument for choosing one joint angle definition over another, any conclusion or interpretation drawn from this analysis would be quite arbitrary. In fact, when the data is represented in the space of hand coordinates shown in Figure 1E, any directional structure of its distribution disappears completely. (For simplicity of exposition, a bivariate Gaussian distribution with equal variance in both directions and mean on the target line was assumed.) Any claim that this data variability illuminates how the CNS organizes its control of behavior would be specious at best.

To check this qualitative impression with quantitative analysis, we randomly selected 100 data points from the set of 500 and conducted UMC analysis. The random selection was repeated 10 times with replacement and the same analysis was performed. Following Scholz and Schöner (1999), we report the results as the ratio of parallel over orthogonal variance. Table 1 summarizes the means and standard errors of the results. Evidently, the UCM ratio is very different for the two coordinate choices. For comparison, we also analyzed TNC components for both coordinate choices, which will be described below.

**Table 1:** Results of UCM and TNC analyses of the data shown in Figure 1C and 1D (absolute and relative joint angle coordinates).

	UCM analysis	TNC analysis		
	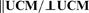 (%)	Tolerance cost (mm)	Noise cost (mm)	Covariation cost (mm)
absolute	101.6±3.7	0.48±0.09	5.91±0.38	0.08±0.01
relative	227.0±8.7	0.48±0.09	5.91±0.38	4.25±0.28

Entries show the mean ± standard error of each measure based on 10 independent random samples of 100 points each drawn with replacement from the 500 hypothetical data points. Both the UCM results and Covariation cost are sensitive to the choice of coordinates. In contrast, Tolerance cost and Noise cost are unaffected.

Despite this sensitivity the core idea remains appealing: a preferential distribution of performance variability along do-not-matter directions suggests skilful CNS control. With a view to overcoming these weaknesses, we identify two reasons why this analysis is sensitive to coordinates: it relies on orthogonality; and it relies on anisotropic distribution of data.

### Orthogonality

**Figure 2:**
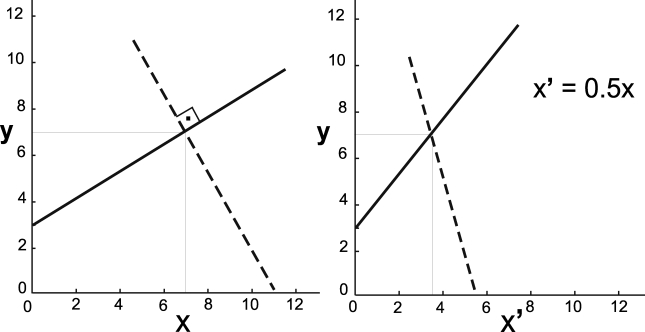
Illustration how orthogonality depends on the chosen scale of the coordinates. Simple multiplication of the x-axis units with a constant factor 0.5 distorts the orthogonal direction in the original *x*,*y* space.

As reviewed above, central to the UCM approach is the projection of performance variability into the nullspace of the Jacobian matrix and its orthogonal complement. Unfortunately, orthogonality is exquisitely sensitive to the coordinates of the space within which it is defined. Figure 2 illustrates this fact: simply changing the scale of the abscissa (multiplying by a constant) changes an orthogonal intersection of straight lines to an intersection at an angle.

Is this a realistic concern? An argument might be made that joint angles should always have the same units. While a joint space with homogenous units is physically reasonable, the physical identity of angular displacements of different joints does not guarantee that they are represented as identical in the CNS. One hypothetical alternative is that joint angles may be represented in the CNS scaled by their range of motion. Different joints have different ranges of motion such that 30 degrees may constitute 100% of maximum range in one joint but only 50% in another. If this were the case, orthogonal directions in a physically-defined space would no longer be orthogonal when transformed into a space that is meaningful to the CNS.

The fundamental problem is that orthogonality requires a *metric* (a function defining the distance between two points in a space) yet plausible coordinates of CNS representations may not admit a metric. For example, Todd and colleagues present convincing evidence that visual space does not have a metric structure [18], [19]. Behavioral evidence of an equivalent finding for the motor system was provided by Fasse and colleagues who showed that at least some aspects of human perceptual-motor behavior do not admit a metric structure [20]. To underscore the behavioral evidence, if joint angles are perceived with respect to an external spatial reference, as reported by Soechting and Ross [21] then they *cannot* admit a metric because finite rotations with respect to an extrinsic spatial reference do not commute and hence violate one of the fundamental requirements to define a space with a metric. In sum, an assumption of orthogonality requires far more structure than may reliably be assumed of CNS representations and hence does not provide a sound basis from which to study CNS control.

### Anisotropy of the Data Distribution

**Figure 3:**
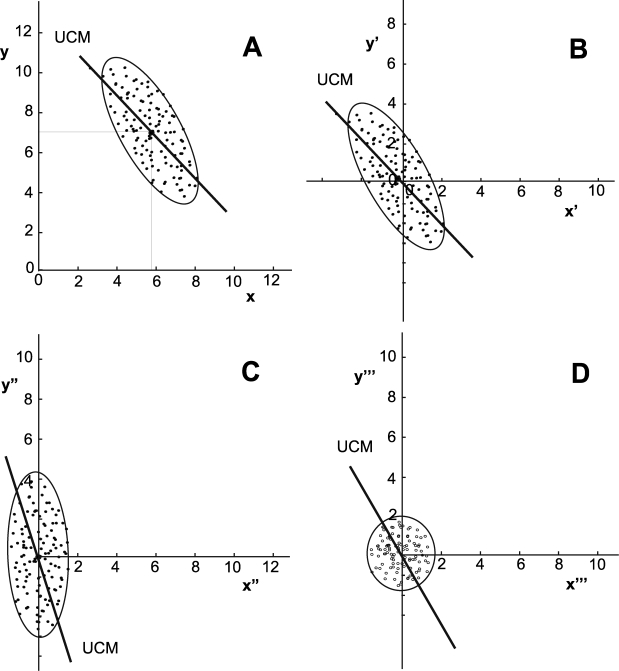
Illustration how simple matrix operations transform an anisotropic data set into an isotropic data set. **A**: Data set with covariance and a mean defined at 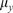 and 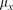; the linearized solution manifold, UCM, is shown by the solid line. The data show anisotropy in alignment with the UCM. **B:** A linear shift centers the data to the origin of new coordinates, *x′*, *y′*. **C:** A rotation of the data aligns the major axis of the data with one coordinate to determine the new coordinates *x″*,*y″*. **D**: A final operation shrinks the major axis to obtain an isotropic distribution in the new coordinates, *x″′*,*y″′*.

As summarized above, the UCM method tests an experimental data distribution for direction-dependent or anisotropic variance in order to assess support for its hypotheses. However, anisotropy of a covariance matrix can *always* be eliminated by a sequence of coordinate transformations (see Text S1). Figure 3 illustrates this basic fact. Panel A shows a hypothetical data distribution in *x*, *y* space. The ellipse denotes the covariance of this distribution. The solid line represents a hypothetical uncontrolled manifold that cuts through the distribution at an angle slightly different from the major axis of the ellipse. Applying simple vector addition, this distribution can be shifted so that its mean coincides with the origin of new coordinates denoted by *x′*, *y′* (Figure 3B). With a simple coordinate rotation, the major axis of the distribution can be aligned with one of the coordinate axes, now defined as *x″*, *y″* (Figure 3C). Finally, re-scaling these axes so that the major and minor axes of the ellipse are equal yields new coordinates, now denoted by *x′″*, *y′″*, in which the covariance is completely isotropic (Figure 3D). In sum, for any data distribution, alternative coordinates can *always* be found in which the directional dependence of variance disappears.

If analysis of covariance matrix anisotropy is applied to seek evidence for the coordinates of CNS control, then this line of argument is troublesome. A set of coordinates is assumed for the execution variables; a particular form of data anisotropy is presented as evidence that those coordinates are, in fact, used by the CNS—but the anisotropy of the data is completely determined by the coordinates initially assumed. There are always alternative coordinates in which the data anisotropy may be eliminated. There are even alternative coordinates in which data anisotropy may be constructed to argue for the opposite conclusion. Unless the coordinates of execution space are objectively known *a priori*, the presence of data anisotropy cannot serve as evidence of control.

These concerns are by no means confined to the UCM method. Covariance-based analyses of variability are in widespread use. They include principal component analysis, factor analysis, ridge regression, proper orthogonal decomposition, linear discriminant analysis, Karhunen-Loève or Hotelling transform, the Isomap method, and non-negative matrix factorization [22], [23], [24]. Most of them depend similarly on assumptions about coordinates. In the study of motor control, covariance-based analysis has been used to infer synergies underlying multi-dimensional motor behavior [12], [25], [26] and many others). For example, Cusumano and Cesari [27] proposed an analysis of variability with respect to a Goal-Equivalent Manifold (GEM, formally equivalent to the UCM). While some details of the GEM method differ from the UCM method (e.g., the use of a singular-value decomposition), most steps are similar—most importantly the analysis of a covariance matrix with respect to the nullspace of a Jacobian matrix. Although the authors do not interpret their findings as identifying the coordinates of CNS control, their results similarly rely on orthogonality and data anisotropy and hence are exquisitely sensitive to the coordinates assumed for the analysis.

In the same vein, Todorov and colleagues have developed a stochastic optimal feedback control framework where, again, variability in the execution of redundant tasks is evaluated to adduce evidence of feedback control [16], [17]. Deviations from a desired target behavior that are preferentially distributed along do-not-matter directions are taken as evidence of optimal control following a Minimum Intervention Principle. As before, although this is an intuitively appealing idea and uses sophisticated mathematical tools, experimental evidence derived from analysis of a covariance matrix is fundamentally sensitive to assumed coordinates. Unless the coordinates of control are objectively known *a priori*, anisotropy of a covariance matrix cannot provide reliable evidence.

### The TNC Method and the Solution Manifold

Can alternative methods be formulated which are less sensitive to coordinates? Sternad and colleagues introduced the so-called TNC analysis (Tolerance – Noise – Covariation) with the goal of quantifying skilled performance and how it changes with practice [28], [29], [30], [31]. In TNC analysis, variability in performance is parsed into three components: Tolerance (or T cost) quantifies to what degree variability is in regions of execution space that are tolerant of error; Noise (or N cost) quantifies to what degree random variation affects performance; Covariation (or C cost) quantifies to what degree covariation among execution variables takes advantage of the structure of the manifold of solutions. The principal goal of this method is to afford a more differentiated view of how the acquisition of skill not only decreases variability but also takes advantage of the structure of the task. Adjusting execution variability affords three conceptually at least different routes to improve performance, and T cost, N cost, and C cost are measures of these three distinct strategies.

In addition, the TNC method differs from those discussed above in one key aspect: instead of evaluating the structure of a covariance matrix in the space of *execution* variables, the quantification of variability is performed in the space of the *result* variable(s) [29], [32]. In a well-posed task, result variables typically have an unambiguous physical meaning and are expressed in a space with a natural, physically-meaningful metric. For that reason, a suitably formulated analysis of performance variability in the space of result variables may be insensitive to their coordinates. In the following we assess the sensitivity of the TNC method presented in Cohen and Sternad (2009) to the experimenter's choice of coordinates.

TNC analysis begins with a model of the task, unambiguously described in physical variables that are measured. For example, analyzing a challenging throwing task where a subject throws a tethered ball around a central post to hit a target, execution is fully determined by two variables (for a detailed description of the task see [28]. They may be the angular position and velocity of the hand at the moment of release of the ball, though other variables may also be chosen (see below). Together they define a two-dimensional *execution space*, *X*. Given these execution variables, the subsequent ball trajectory—and hence the outcome of any throw—is fully determined from elementary mechanical physics. The result of any particular execution is an error, specifically, the closest approach of the ball to the target. It defines a one-dimensional *result space*, *R*. The task is redundant as multiple combinations of the two execution variables yield the same result; a function 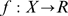 is readily identified which describes a “many-to-one” map from execution space into result space.

**Figure 4:**
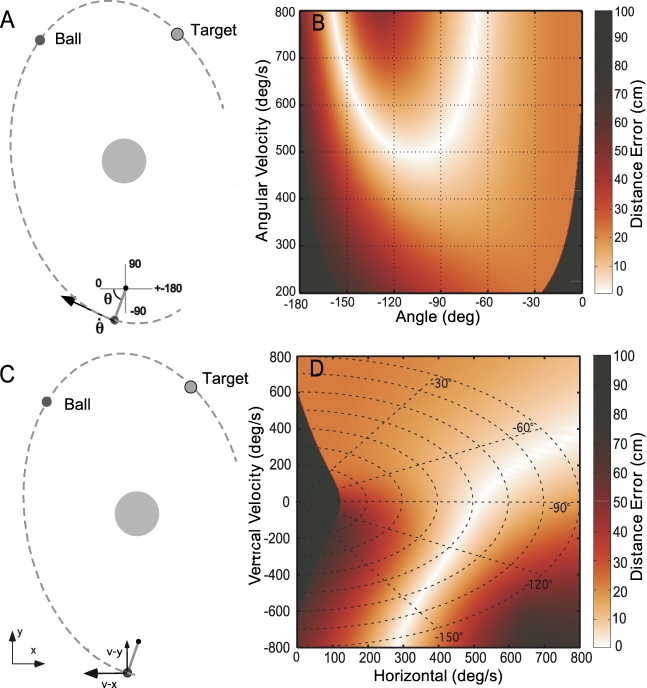
Skittles task and result function in two different coordinates. **A**: Top-down view of the work space of the skittles task with the manipulandum at the bottom. The dashed line denotes the trajectory of the ball as it goes through the target with zero error. The center circle is an obstacle to ensure non-trivial ball releases. The two execution variables of the manipulandum are defined in polar coordinates, angle and angular velocity at release, 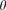 and 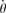. **B**: Three-dimensional rendering of the corresponding execution and result space. The grey shades code the error magnitude with white showing zero error and darker grey shades increasing non-zero error. **C**: The two execution variables defined in Cartesian coordinates, *v-x* and *v-y*. **D**: Three-dimensional rendering of the corresponding execution and result space.

Perfect execution of this task with zero error defines the solution manifold shown in white in Figures 1C, 1D and 4. Non-zero errors are defined by the result function *f* and determine a landscape—an elongated “valley” with the solution manifold as its bottom—with error magnitudes expressed in colors with darker denoting larger errors (for details see Cohen & Sternad, 2009). The solution manifold (and, indeed, the entire result function) is highly nonlinear because the tether pulls the ball towards the central post, giving it a curved flight path. To be strictly correct, the nonlinear result function is itself a 2D manifold in the 3D space formed by the composition of the result and execution spaces, 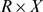. However, to facilitate comparison with related methods, in this paper we reserve the term “manifold” for the zero error result (the solution manifold) though, technically, it defines a 1D sub-manifold of the 2D result function. The solution manifold, SM, is formally equivalent to the UCM and the GEM. Note that the existence of a solution manifold (UCM or GEM or SM) with a dimension of one or higher is a requirement for a task to be redundant.

An important point is that the definition of the solution manifold is independent of any assumptions about the coordinates of execution space. *It is always possible to establish a complete equivalence between the solution manifolds expressed in any two alternative choices for execution space coordinates*. The reason is simple: if we visualize the result function as a 2D landscape in 3D space, the solution manifold is always at the “bottom of the valley”. While the curve corresponding to the bottom of the valley may appear different with different coordinates of execution space, it always corresponds to zero result. This is illustrated in Figure 4, which depicts the result function of the skittles task for two plausible choices of the execution variables: the angular position and velocity of the hand at the moment of release of the ball, which may loosely be termed polar coordinates (Figure 4A); and two orthogonal components of the velocity of the hand at the moment of release, which may be termed Cartesian coordinates (Figure 4B). The corresponding result functions are shown in Figures 4C and D, respectively. Because the relation between these two coordinate frames is nonlinear, each result function is a distorted copy of the other and the solution manifold traces a different curve in each space. However, in both cases, the solution manifold corresponds to identically zero result—it is at the bottom of the valley. Of course, because the UCM, GEM and SM are equivalent, all enjoy this property. However, the UCM and GEM methods confine their variability analysis to execution space and take no advantage of this fact.

The TNC method analyzes observed performance in the context of the result function and distinguishes several related aspects of imperfect performance. First, it is commonly observed that subjects do not use the entire solution manifold, even though all combinations of execution variables that lie on it yield equally perfect performance. Instead, performance attempts tend to be clustered around a preferred location on the solution manifold (Cohen & Sternad, 2009), most likely because different locations have different tolerance of errors. *Tolerance cost* provides a measure of how observed performance exploits error tolerance by shifting the observed data distribution to different locations in execution space and evaluating the greatest ensuing improvement in average result. Second, subjects are not only *inaccurate* but also *imprecise*. *Noise cost* provides a measure of how this random scatter around the mean execution affects performance. Noise cost is calculated by shrinking the set of data incrementally and uniformly towards its mean in execution space. The greatest improvement in average result that ensues is taken as Noise cost. Third, even if variable errors are not reduced, they may be structured to advantage. *Covariation cost* provides a measure of how observed performance capitalizes on the structure of the solution manifold. It is calculated by recombining observed data in execution space and evaluating any improvement in average result. The important point for present purposes is that these three features of performance variability are estimated in result space, in units of the result variable. They are expressed as costs indicating how much observed performance could have been improved by an appropriate change of tolerance, noise and covariation.

### Curvature of the Solution Manifold

One obvious reason to prefer some locations on the solution manifold over others is the sensitivity of the result to variability (or, equivalently, tolerance of error). This is determined in part by the mechanical physics of the task, expressed as the curvature of the result function. Figures 4C and 4D show the result function of the skittles task (depicted as a plan view of a curved valley). Due to the nonlinear mechanics of the task, the immediate neighborhood of the solution manifold has different curvature at different positions, making some locations more tolerant of errors than others. Note that all locations on the solution manifold have identical height and there is no global minimum or “best” location based on error alone. It is the “width” of the valley that varies with location (or, equivalently, how close its bottom is to being flat). Looking beyond the one-dimensional solution manifold to the many-dimensional result function opens up additional ways to quantify the consequences of variability, such as to assess the effect of curvature on error tolerance.

Remarkably, important features of the result function's curvature are *completely independent of any assumptions about the coordinates of execution space*. As discussed above, the solution manifold itself is independent of coordinates. In addition, if the result function smoothly maps execution space into result space, the Hessian matrix (a matrix of second partial derivatives) of that map evaluated at any point determines its curvature at that point. Because the result function is real-valued and continuous, its Hessian matrix is real-valued and symmetric and has real eigenvalues. The eigenvalues determine the maximum and minimum curvatures (known as principal curvatures) of the result function. However, the result (error) is identically zero at all points on the solution manifold and is positive at all other points. Therefore we may deduce that: (i) the smallest principal curvature is always zero; (ii) the largest principal curvature is always non-negative. As a result, the Gaussian curvature (the product of the principal curvatures) of the result function is identically zero at all points along the solution manifold. *For any coordinates that may be used for execution space*, *the Gaussian curvature on the solution manifold is zero*.

### Coordinate Sensitivity of TNC Analysis

These geometric considerations justify a guarded optimism that methods based on analyzing subject performance in the context of a result function may enjoy less sensitivity to the coordinates assumed for execution space. Does empirical evidence support this conjecture? While the result function for the skittles task is derived from simple mechanical physics, the same physical principles can be expressed in many alternative coordinate systems. One reasonable candidate is the “polar” coordinate frame used above (and detailed in Figure 4A and 4C)—angle and angular velocity at the moment of release. An equally reasonable alternative is the Cartesian components of linear velocity at the moment of release (detailed in Figure 4B and 4D). Either pair of variables fully determines the subsequent ball trajectory and the ensuing error at the target. Note that the relation between these two coordinate systems is significantly more challenging than the simple linear transformation between absolute and relative joint angles in the hypothetical example of Figure 1.

**Figure 5:**
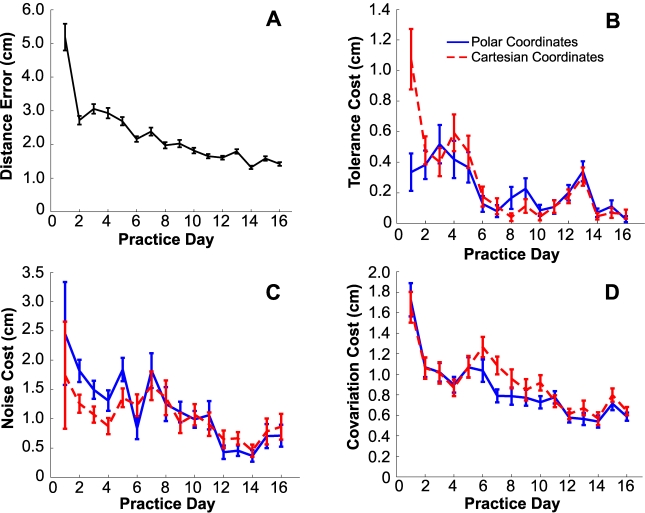
Comparison of Tolerance, Noise and Covariation costs in two coordinate frames (data of one subject from Cohen & Sternad, 2009). **A**: Average distance error plotted across 16 days of practice gives evidence of performance improvement. The error bars denote standard error. **B**: Average Tolerance cost over the 16 days of practice. **C**: Average Noise cost over the 16 days of practice. **D**: Average Covariation cost over the 16 days of practice. In B, C and D the dashed lines denote polar coordinates, the solid lines denote Cartesian coordinates. Error bars denote standard deviations. Variance was computed using a bootstrap procedure: 100 samples from the total set of 180 data were randomly drawn (with replacement) and the costs were calculated; this procedure was repeated 100 times with different samples. In either coordinate frame the results show qualitative agreement as discussed in the text.

To assess the sensitivity of TNC analysis to this coordinate transformation, we calculated Tolerance, Noise, and Covariation costs for a particular set of experimental data expressed in polar and Cartesian coordinates. The specific data were taken from a study by Cohen and Sternad (2009) and represent one expert subject practicing the skittles task for 16 days with 180 throws on each day. The study was approved by the Institutional Review Board of Pennsylvania State University (IRB#: 16237). As expected, a pronounced learning curve is observed in the distance error data (Figure 5A). The daily average of the three different costs together with their standard deviations are displayed in Figures 5B, 5C and 5D, respectively. The cost calculations performed in polar and Cartesian coordinates are shown in dashed and solid lines respectively.

The greatest influence of coordinates is on Tolerance cost on day 1, when the error is also highest. By day 2 this influence has largely disappeared and the error has declined dramatically (the largest day-to-day performance improvement observed). As is typical, this subject's initial execution attempts on day 1 were widely scattered, covering a large range of execution space. Over this range, the relation between the different coordinates is highly nonlinear. On subsequent days the execution attempts were more tightly clustered covering a smaller range of execution space (as evidenced by the decline of Noise cost over the first few days). Over this narrower range, the relation between the different coordinates is closer to linear. As we show below, while Tolerance cost may be affected by a nonlinear transformation of coordinates, it is completely insensitive to any linear transformation of coordinates. More tightly clustered execution attempts yield smaller Noise cost; the transformation between coordinates becomes progressively closer to linear; and the choice of coordinates has progressively less influence on the analysis. Indeed, from about day 10 onwards, Tolerance costs are effectively indistinguishable in the different coordinates. Noise costs and Covariation costs are also remarkably similar.

In sum, although the choice of coordinates produces some early quantitative differences, the qualitative trends for each of the three costs are remarkably similar and the quantitative differences vanish as skill improves. Although these two coordinate frames are substantially different and nonlinearly related, those features of the data analysis that convey the most potential meaning for studies of motor coordination and learning—the order-of-magnitude differences, the overall trend over successive days, the rank-ordering of the costs—are largely unaffected.

### Influence of Coordinate Transformations

Is this insensitivity to coordinates a general property of TNC analysis or a fortuitous outcome of analyzing a “favorable” data set? In the following we consider each part of TNC analysis in turn.

#### Covariation cost

To calculate Covariation cost, pairs of observed data points are re-combined by exchanging one of their coordinate values. This operation has no effect on the marginal distributions of the observations but it may improve the result. A search procedure finds the pairings that yield the minimum average result. The difference between the original average result and the minimum is Covariation cost (for a more detailed description of calculations see Cohen & Sternad, 2009). This procedure provides a measure of the alignment (or misalignment) of a subject's performance attempts with the solution manifold. Note, however, that unlike UCM or other covariance matrix factorization methods, it does not require the data distribution to be anisotropic. Neither does it depend on orthogonality, as the measure of closeness to the solution manifold is determined by comparing averages in result space.

Unfortunately, because marginal distributions are projections of the observed data distribution onto the coordinate axes, this Covariation cost is sensitive to a rotation of the coordinate axes of execution space. To illustrate, suppose a particular distribution of observations is clustered in a region where the solution manifold is only mildly curved (which is often the case). If the coordinate axes are rotated so that the roughly-straight section of the solution manifold is parallel to the coordinate axes, then re-combining pairs of data points will have minimal effect on the average cost. In this example it would always be possible to find a coordinate frame in which Covariation cost was essentially zero—no matter what the Covariation cost was in the originally chosen coordinates. This problem is illustrated in the hypothetical example of Figure 1. In either coordinates, the solution manifold is only mildly curved. In relative joint angles a substantial Covariation cost is observed, but in absolute joint angles it is many times smaller (see Table 1, column 4).

**Figure 6:**
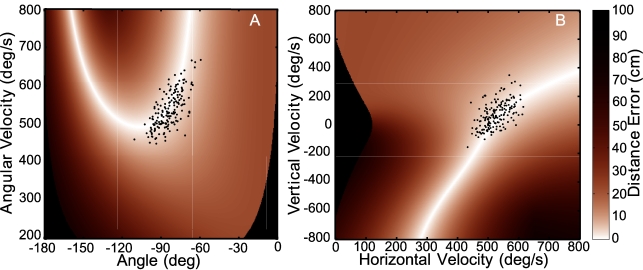
Exemplary data in polar (A) and Cartesian (B) coordinates. The data are one block of 60 performance attempts on the second day of practice by the same subject shown in Figure 5. In both coordinate systems the data cluster in a region where the solution manifold (white) is mildly curved and approximately diagonal.

Although this measure exhibits an undesirable sensitivity to coordinates, the origin of that sensitivity is evident. Consequently, those conditions under which sensitivity to coordinates is minimal are readily identified: provided the solution manifold has a predominantly diagonal disposition in the region of execution space occupied by the data, the particular choice of execution coordinates will make little difference. That is consistent with the observation that Covariation cost shows a similar trend to decrease with practice in either polar or Cartesian coordinates—in both of the coordinates considered, the observed executions occupy a region of the solution manifold which is predominantly oriented diagonally (Figure 6). Of course, a measure that would be completely independent of the choice of coordinates is clearly desirable. One possible way that might be accomplished is considered in the discussion.

#### Noise cost

Noise cost is computed by progressively contracting the distribution of performance attempts in execution space towards its mean in a series of small steps. At each step, the average result is evaluated and the greatest improvement in average result that ensues is Noise cost. In general, the distribution of a set of attempts may vary with the coordinates assumed for execution space. For example, if two coordinate frames are nonlinearly related (e.g., polar and Cartesian coordinates), then the mean of the distribution in the two coordinates will, in general, correspond to different values of the result. Theoretically, then, Noise cost depends on coordinates but in practice the sensitivity may be small. In Text S2 we present a brief analysis showing that if the result function curvature is small in the region occupied by experimental data, then Noise cost is minimally affected by a linear transformation of execution coordinates. In other words, if the curved surface of the result function may be approximated competently by a tangent plane in the region occupied by the data, then a linear coordinate transformation will have no material effect on Noise cost. In that case the Noise cost calculation would be indifferent to a change from absolute to relative angles (see Figure 1) that may profoundly affect covariance-based analyses (see Table 1, column 3).

This argument may be extended to nonlinear coordinate transformations. If they, too, have sufficiently small curvature in the region occupied by experimental data, Noise cost will be minimally affected. Pragmatically, experimental observations are often clustered in a small region of execution space. Furthermore, as subjects acquire skill they tend to cluster their performance even more closely. Consequently, we may expect Noise cost to be at worst weakly sensitive to coordinates, and become progressively less sensitive as subjects acquire skill. This is consistent with the insensitivity of Noise cost to polar or Cartesian coordinates shown in Figure 5.

#### Tolerance cost

Tolerance cost is computed by translating an observed set of performance attempts to a new location in execution space without changing its distribution and evaluating the corresponding average result. This is repeated for translations to all points on a grid covering an experimentally reasonable range of execution space to find the translation that yields the minimum average result. The difference between the original average result and the minimum is Tolerance cost. In Text S2 we present a brief analysis showing that Tolerance cost is completely unaffected by *any* linear transformation of execution coordinates. This is a large class of transformations; it includes rotation, dilation, contraction and shearing of the coordinate axes. In particular, the Tolerance cost calculation is indifferent to a change from absolute to relative angles (see Figure 1) that profoundly affects covariance-based analysis (see Table 1, column 2).

As with Noise cost, a nonlinear transformation of coordinates—such as between the polar and Cartesian coordinates—may affect Tolerance cost; a translation of the data distribution that does not change its shape in one coordinate frame may change its shape in another frame. However, as with Noise cost, if the data occupy a region in which the curvature of the nonlinear coordinate transformation is sufficiently small, Tolerance cost is minimally affected. This is consistent with the insensitivity of Tolerance cost to polar or Cartesian coordinates shown in Figure 5.

## Discussion

Redundancy in the execution of a given motor task presents alternatives to the central nervous system and affords an opportunity for sophisticated control. If some elements of the neuromuscular system are no longer available, such as due to injury, the system can maintain its functionality; it is robust. Given that complex multi-level systems typically have noise, redundancy also provides ways to cope with this noise and channel it into directions that have minimal effect on achieving the task goal. Hence it is reasonable to hypothesize that skilled performers take advantage of this redundancy and align their actions with the solution manifold corresponding to a given task goal, i.e. the space in which noise and variability have little or no effect on the end result. Consequently, analysis of variability in such redundant tasks promises insight into the CNS control system. For example, evidence that behavior adapts to take advantage of the solution manifold in execution space sheds light on what the CNS controls—not execution variables per se but combinations thereof. Further, the decomposition into three factors which change with different time histories, as shown in the TNC analysis of the exemplary data, provides insight about routes for change that are otherwise not visible.

Though numerous studies have tried to pursue these questions via analysis of variability, a problem arises when covariance is the only basis of this line of investigation. Specifically, we showed that methods based on analysis of a covariance matrix are exquisitely sensitive to the coordinates within which the analysis is conducted. This is no small consideration as covariance-based methods are ubiquitous in movement neuroscience and other disciplines. However, their sensitivity to assumed and measured coordinates confers an uncomfortable arbitrariness on their outcome and motivates the work presented here—an initial attempt to assess the coordinate dependence of alternative methods of analysis.

One way to cut this Gordian knot might be to find out in advance which coordinates are relevant for the nervous system. Unfortunately, in behavioral research this is unlikely; the variables that are meaningful to the CNS are typically not known *a priori*. Indeed, to identify which variables the CNS may control is one of the central questions of motor neuroscience.

An alternative is to rely on statistics of higher order than covariance. An example is the so-called “infomax” algorithm introduced by Bell and Sejnowski [33]. It is a self-organizing learning algorithm that maximizes entropy of the output of a single-layer neural network. The authors reported that it converges to independent component analysis (ICA) of the input signals and accomplishes “blind source separation” (i.e. teasing apart independent sources of a composite signal without *a priori* knowledge of the source characteristics). However, in a more recent paper Xi, Chicharo, Tsoi, and Siu [34] demonstrated that the infomax algorithm is not able to separate signal sources if the data are not first de-correlated. Furthermore, the authors report that de-correlation alone is often sufficient for blind source separation. Because de-correlation depends only on the data covariance matrix (it corresponds to the first two steps illustrated in Figure 3, panels A through C), these results indicate that the infomax algorithm is also sensitive to the experimenter's assumed coordinates.

The infomax algorithm is only one of many approaches to ICA. A general survey is provided by Hyvärinen and Oja [35] who emphasize that a non-Gaussian data distribution is essential. Consequently, a change of variables that “distorts” an original data distribution so that it becomes Gaussian will disable ICA, whether performed by infomax or any other algorithm. However, for continuous univariate data distributions it is always possible to identify a (nonlinear) change of variables such that the transformed data follows a Gaussian distribution; for multivariate distributions a proof is more challenging. While ICA may afford advantages in particular cases, the central problem remains: the analysis depends on the experimenter's choice of coordinates.

Another way to circumvent the problem of coordinate sensitivity may be to ground the analysis in that set of coordinates which renders variability of the initial performance isotropic or, equivalently, focus only on the change of anisotropy. Several studies have aimed to identify functional synergies and their development with practice or recovery after injury in this way [12], [36], [37], [38]. If the coordinates are (re-)defined such that the initial or reference data have an isotropic covariance matrix, then any changes will be revealed as increases in the anisotropy. While this would appear to address the immediate difficulty, this coordinate set will likely differ between individuals, rendering comparisons between individuals problematic or even impossible. It is not even clear whether it would support comparison between the same individual's performance on different occasions or on different tasks. Essentially, the choice of the reference observation used to define the initial coordinates re-introduces the problem of arbitrariness; it would be equally appropriate to choose the coordinates for isotropy at any other reference point.

### Towards Coordinate-Independent Methods

In physics it is generally expected that descriptions of natural phenomena should not depend on an arbitrary choice of the coordinates in which the descriptions are cast. This principle has thus far received little consideration in movement neuroscience, which is surprising given that neuroscience similarly seeks fundamental descriptions of the function of the neuromuscular system. Because the tensor calculus is one of the classical methods to formulate analysis independent of coordinate frames, the “tensor theories” of sensory-motor transformations within the CNS (proposed by Pellionisz and Llinas [39]) might appear to address this matter. Unfortunately, as detailed in the review by Arbib and Amari [40], their use of tensor calculus was at best metaphorical and could not achieve the required independence of coordinate frames.

Sensorimotor transformations within the CNS might alternatively be approximated by a weighted combination of suitable basis functions, an approach that could plausibly be implemented by e.g. three-layer networks of neurons. Soechting and Flanders (1992) point out that “…activity in intervening (hidden) layers need not be in any frame of reference…”. Pouget and Sejnowski [41] propose that single neuron responses serve as basis functions which “have the advantage of not depending on any coordinate system or reference frame.” The substance of this statement is that the different nonlinear functions required to represent the same sensory event or motor response in different reference frames may be approximated as different linear combinations of the same set of basis functions. As a result, the different representations are related by linear transformations. However, this does not achieve the required independence of coordinates that we seek. The “relative” and “absolute” joint angles considered in the example of Figure 1 are related by a linear transformation, yet the difference between them profoundly affects an analysis of the distribution of experimental observations.

An important distinction should be made between the coordinates of a putative *internal* neural representation and the coordinates of *external* observations of behavior that may be used to infer neural processes. Because the complexity of the central nervous system and the limitations of available measurements create boundless opportunities for confusion, it seems prudent (perhaps even mandatory) to seek descriptions and analysis techniques that are minimally affected by an investigator's choice—however sensible—of measures and coordinates. If that should prove to be impractical, it is at least necessary to understand how a change of coordinates may affect the conclusions drawn; this was the primary motivation for the study reported here.

Given the difficulties inherent in any method based on covariance, we considered an alternative analysis of data structure, the TNC method. One of its distinguishing features is that quantitative assessment of structure in *execution variability* is evaluated *in the space of the result* (see Müller and Sternad, 2009). The key point is that while different coordinates of execution space may be chosen, the result does not change. In the example cited above, the result space was one-dimensional (the distance of closest approach to the target) but that is not essential. Though the clarity of one-dimensional measures of task success affords substantial advantages, multi-dimensional result spaces could be envisioned. Alternative result measures are discussed in Müller and Sternad (2003). However, in any unambiguously defined task, the result space should admit a natural metric so that any improvement (or decline) in performance could be identified unambiguously. For example, in the hand space depicted in Figure 1E, distance is naturally quantified by the usual Euclidean metric. Therefore, orthogonality is uniquely defined in hand space. In addition, physical distance is invariant under changes of hand coordinates. If the experimenter chose to use, say, polar coordinates to quantify hand position, a different well-defined metric (obtained by suitably transforming the Euclidean metric) applies to these coordinates. In some tasks it might be advantageous to define a result space whose elements were the complete time-histories of performance attempts. To be defined unambiguously, this result space should be an infinite-dimensional Hilbert space. Though we anticipate no fundamental barriers to dealing with these more challenging cases, their analysis is deferred.

Provided the result space has a well-defined metric, any analysis of behavior *confined* to result space may be made completely independent of the choice of its coordinates. For example, though Lacquaniti and Maioli (1994b) arrive at their main result (planar co-variation of joint angles) by principal component analysis in the space of joint angles (which is sensitive to the choice of joint angles) this may be interpreted in terms of CNS control of leg length and orientation (Maioli & Poppele, 1991). While the existence of any metric for any configuration space of joint angles is debatable, there is a natural choice for the location of the forepaw or hindpaw relative to the shoulder or hip: the Euclidean distance between the proximal joint and its distal support. Insofar as conclusions are drawn from observations of minimal variability of foot trajectory in space [42], they are also insensitive to the choice of spatial coordinates. However, it would be difficult to extract convincing evidence of synergies or how the CNS may solve the problem of controlling redundant degrees of freedom from any analysis that is confined to result space alone. For that reason TNC analysis maps execution space onto result space.

Sensitivity to coordinate transformations is only brought about by operations that are performed in execution space. In TNC analysis, those operations consist of translation, uniform shrinking, and re-combination of the observed data. Linear transformations of the execution coordinates do not affect the translation used to calculate Tolerance cost. For example the change from absolute to relative joint angles, which profoundly affected UCM analysis, makes no difference whatsoever. This is not to say that Tolerance cost is indifferent to all coordinate changes; it is clearly affected by nonlinear coordinate transformations. Its sensitivity is determined by how much a nonlinear coordinate transformation departs from linearity over the region of analysis. Sufficiently “gentle” transformations (i.e., those sufficiently close to linear) will have little influence. We presented empirical evidence suggesting that a nonlinear transformation between polar and Cartesian coordinates has minimal effect. Nonetheless, it would be advantageous to develop a (revised) measure of tolerance that was insensitive to coordinates. That is a topic of ongoing investigation.

Linear transformations of the execution coordinates are expected to have little effect on the shrinking operation used to calculate Noise cost. In this case, the sensitivity to coordinates will be determined by the curvature of the result function over the region occupied by the data. Again, empirical evidence suggests that a nonlinear transformation between polar and Cartesian coordinates has little effect.

As described above, the re-combination operation used to calculate Covariation cost is fundamentally sensitive to rotation of the coordinate axes [43], [44]. Even so, this measure has some singular merits: it is not affected by the core weaknesses of methods based on covariance matrix factorization because (i) it makes no use of orthogonality, and (ii) it does not require anisotropic distribution of the data. It is therefore worth considering how it might be improved.

### Revised Covariation Cost

**Figure 7:**
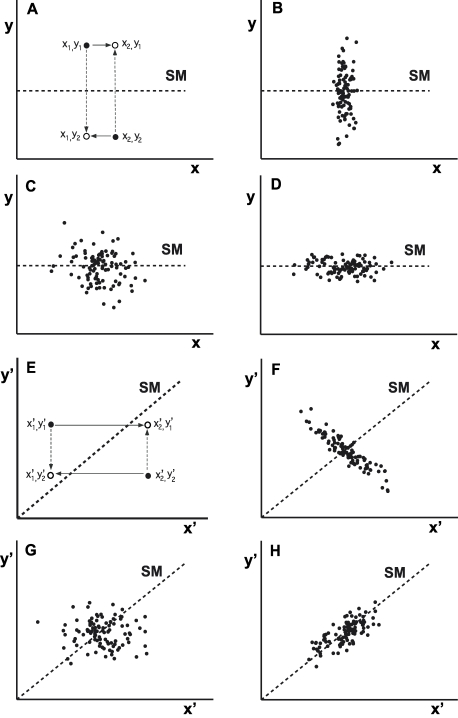
Schematic illustration of the Covariation cost calculation and how it depends on the solution manifold orientation. **A–D**: If the solution manifold is oriented parallel to the *x*-axis, exchange of coordinates of two exemplary points *x*_1_,*y*_1_ and *x*_2_,*y*_2_ does not lead to an improvement in average result. Panels B, C, and D show that this holds for three different distributions. **E–H**: In contrast, if the solution manifold is oriented diagonally, exchange of coordinates of two exemplary points *x*_1_,*y*_1_ and *x*_2_,*y*_2_ leads to an improvement in average result. This improvement will be greatest if the data distribution is aligned along a direction different from the solution manifold (F); intermediate if it exhibits no apparent alignment (G); and close to zero if it is well-aligned with the solution manifold (H).

As outlined previously, the reason Covariation cost is sensitive to coordinates is clear. The difficulty is illustrated in Figure 7. Panels A and E depict how any two observations *x_1_*, *y_1_* and *x_2_*, *y_2_* (schematically shown as two filled dots) may be re-combined to produce new data *x_1_*, *y_2_* and *x_2_*, *y_1_* (shown as open circles). Exchanging their *x*-coordinates (or *y*-coordinates) does not change the marginal data distributions but might change the corresponding results. If the solution manifold and adjacent lines of constant result are (approximately) straight and aligned parallel to one of the coordinate axes as in Figure 7A–D, this re-combination will have no effect. As a result, Covariation cost will be (approximately) zero. Figure 7B–D illustrates this for schematic data with a distribution that is completely misaligned with the solution manifold (Figure 7B), or exhibits no apparent alignment (Figure 7C), or is well-aligned with the solution manifold (Figure 7D). In contrast, if the solution manifold and adjacent lines of constant result are (approximately) straight but aligned diagonally with respect to the coordinates as in Figure 7E–H, then recombination of *x*- and *y*-elements can substantially improve the average result. The effect will be greatest if the data distribution is aligned along a direction different from the solution manifold (illustrated in Figure 7F), intermediate, if it exhibits no apparent alignment (Figure 7G), and close to zero if it is well-aligned with the solution manifold (Figure 7H).

This suggests an obvious way that Covariation cost may be revised to minimize its sensitivity to coordinates: For any choice of execution coordinates, a rectangular region may be identified that bounds some appropriately large proportion of the marginal data distributions (less than 100% to minimize the influence of outliers). Within that region the best straight-line approximation to the solution manifold may be found. From that information, a rotation of the coordinate axes may be identified to define a new coordinate frame in which the solution manifold approximately intersects opposite corners of the rectangular region containing the (new) marginal data distributions. Provided the solution manifold is mildly curved throughout the region occupied by the data, the recombination procedure described previously will yield a (revised) Covariation cost that will approach zero only if the data is distributed along the solution manifold. Furthermore, this revised Covariation cost will be insensitive to the initial choice of execution coordinates, provided again that the solution manifold is approximately straight throughout the region occupied by the data.

This revised measure of covariation adds a step to the analysis to circumvent problems due to an untoward relation between the coordinate axes (chosen by the experimenter) and the solution manifold (defined by the physics of the task). Essentially this revision recognizes the original weakness and turns it to advantage. Nonetheless, it may not confer complete insensitivity to coordinates. A method to do so is a topic of ongoing investigation.

### Importance of the Result Function

The heart of TNC analysis is identification of a result function. In a well-posed task, the goal is explicit and meaningful and presents an unambiguous, objective benchmark for evaluating performance. Incorporating the result function avoids implicit limitations on an analysis of variability. For example, the result function determines the consequences of both inaccuracy (constant error) and imprecision (variable error) for task performance. In contrast, covariance matrix factorization methods are necessarily performed on deviations around a mean, and must remain silent on the consequences of constant error.

Geometric details of the result function may be particularly informative. Though manifestly true, it may not be obvious that all perfect solutions are not equivalent but may differ in their forgiveness of error. Recognizing this fact is potentially a rich source of new insight into central nervous system control, suggesting new perspectives and hypotheses. For example, it seems reasonable to postulate that actors may “exploit” variability to assess error tolerance. This is consistent with the hypothesis that variability is necessary to explore execution space and find good solutions [45], [46], [47], [48]. This hypothesis may be rendered explicit by observing that variability affords a way to assess the curvature of the result function, and may be testable by offering a way to quantify error tolerance via the result function curvature.

This hypothesis is strongly reminiscent of the concept of “persistent excitation” that is essential for effective adaptive control [49]. An essential point is that, if this hypothesis is correct, the best strategy may *not* be to confine variability in the directions that affect task performance to its irreducible minimum and channel the remainder to the do-not-matter directions. As described above, the curvature of the result function along the do-not-matter directions (the solution manifold) is identically zero, so variability in this direction adds little new information. Instead, tolerance of error depends on directions independent of the do-not-matter directions. Variability in these “do-matter” directions may be essential to identify the best location along the solution manifold at which to cluster performance. Exploration of this possibility is a topic of ongoing investigation.

## Supporting Information

Text S1 Diagonalization of a covariance matrix.
(0.09 MB DOC)

Text S2 How coordinate transformations affect Tolerance and Noise costs.
(0.10 MB DOC)
