# Supplementary material for: Coordinate Dependence of Variability Analysis
Source: PLoS Comput Biol. 2010 Apr 22;6(4):e1000751. doi: 10.1371/journal.pcbi.1000751 (PMC2858681; doi:10.1371/journal.pcbi.1000751)
Supplement: Text S1 — Diagonalization of a covariance matrix. (0.09 MB DOC) [file pcbi.1000751.s001.doc]

# Supporting Information I:

# Diagonalization of a covariance matrix

Denote a set of observations of execution variables as $\left\{\mathbf{x}_{1},\mathbf{x}_{2},\mathbf{x}_{3},...\right\}$ where each observation is a vector $\mathbf{x}_{i}\in R^{n}$. The mean value of the set is $\mathbf{\mu}=E\left\{\mathbf{x}\right\}$ where denotes the expectation operator. The covariance of the set is $\mathbf{C}_{x}=E\left\{\left(\mathbf{x}-\mathbf{\mu}\right)\left(\mathbf{x}-\mathbf{\mu}\right)^{t}\right\}$. In two dimensions, $\mathbf{x}_{i}=\left[\begin{matrix} x_{i} \\ y_{i} \end{matrix}\right]$, $\mathbf{\mu}=\left[\begin{matrix} \mu_{x} \\ \mu_{y} \end{matrix}\right]$ and $\mathbf{C}_{x}=\left[\begin{matrix} \sigma_{xx}^{2} & \sigma_{xy} \\ \sigma_{xy} & \sigma_{yy}^{2} \end{matrix}\right]$. The covariance is a symmetric, real-valued, positive-definite square matrix. It therefore has non-zero, real-valued eigenvalues and orthogonal, real-valued eigenvectors which may be defined by $\mathbf{C}_{x}\mathbf{\varphi}=\lambda\mathbf{\varphi}$. If any eigenvalues of the covariance matrix are identical, orthogonal eigenvectors may still be identified, though they are not unique. Choosing the eigenvectors to have unit magnitude, they may be assembled into an eigenvector matrix $\mathbf{\Phi}=\left[\mathbf{\varphi}_{1},...\mathbf{\varphi}_{n}\right]$ such that $\mathbf{C}_{x}\mathbf{\Phi}=\mathbf{\Phi\Lambda}$ where $\mathbf{\Lambda}=diag\left(\lambda_{1},...\lambda_{n}\right)$ and the eigenvector matrix is orthonormal, $\mathbf{\Phi}^{-1}=\mathbf{\Phi}^{t}$.

Consider the following series of coordinate transformations. First, shift the mean to the coordinate origin $\mathbf{x}'=\mathbf{x}-\mathbf{\mu}$. By definition, the mean of the shifted set is zero, $E\left\{\mathbf{x}'\right\}\{\}=E\left\{\mathbf{x}-\mathbf{\mu}\right\}=E\left\{\mathbf{x}\right\}-\mathbf{\mu}=\mathbf{0}$, hence this coordinate transformation has no effect on the covariance, . Second, consider a transformation $\mathbf{x}''=\mathbf{\Phi}^{t}\mathbf{x}'$. This yields a diagonal covariance matrix and is equivalent to rotating the coordinate axes: , hence $\mathbf{C}_{x''}=\mathbf{\Phi}^{t}\mathbf{C}_{x}\mathbf{\Phi}=\mathbf{\Phi}^{-1}\mathbf{C}_{x}\mathbf{\Phi}=\mathbf{\Lambda}$. Third, consider a transformation $\mathbf{x}'''=\mathbf{S}^{-1}\mathbf{x}''$, where $\mathbf{S}=diag\left(\sqrt{\lambda}_{1},...\sqrt{\lambda}_{n}\right)$. This yields a unity covariance matrix and is equivalent to re-scaling the coordinate axes: , hence $\mathbf{C}_{x'''}=\mathbf{S}^{-1}\mathbf{\Lambda S}^{-t}=\mathbf{I}$, i.e.

$$\mathbf{C}_{x'''}=\left[\begin{matrix} 1/\sqrt{\lambda_{1}} & & 0 \\ & \ddots& \\ 0 & & 1/\sqrt{\lambda_{n}} \end{matrix}\right]\left[\begin{matrix} \lambda_{1} & & 0 \\ & \ddots& \\ 0 & & \lambda_{n} \end{matrix}\right]\left[\begin{matrix} 1/\sqrt{\lambda_{1}} & & 0 \\ & \ddots& \\ 0 & & 1/\sqrt{\lambda_{n}} \end{matrix}\right]=\left[\begin{matrix} 1 & & 0 \\ & \ddots& \\ 0 & & 1 \end{matrix}\right]$$

With this series of transformations it is always possible to define alternative coordinates which *completely eliminate* all directional properties of the covariance matrix.
