# Supplementary material for: Coordinate Dependence of Variability Analysis
Source: PLoS Comput Biol. 2010 Apr 22;6(4):e1000751. doi: 10.1371/journal.pcbi.1000751 (PMC2858681; doi:10.1371/journal.pcbi.1000751)
Supplement: Text S2 — How coordinate transformations affect Tolerance and Noise costs. (0.10 MB DOC) [file pcbi.1000751.s002.doc]

# Supporting Information II:

# How coordinate transformations affect tolerance and noise cost

Denote a result function that maps execution space *X* onto result space *R* by $f:X\to R$ or $r=f\left(\mathbf{x}\right)$, where $r\in R$ and $\mathbf{x}\in X$. Consider a change from initial execution space coordinates $\mathbf{x}=\left[x_{1}\ldots x_{n}\right]^{t}$ to $\mathbf{a}=\left[a_{1}\ldots a_{n}\right]^{t}$ defined by a general transformation $\mathbf{t}:X_{a}\to X$ or $\mathbf{x}=\mathbf{t}\left(\mathbf{a}\right)$. The result function that maps this alternative execution space into result space is defined by simple substitution, $r=f\left(\mathbf{x}\right)=f\left(\mathbf{t}\left(\mathbf{a}\right)\right)=g\left(\mathbf{a}\right)$. In the initial coordinates, the solution manifold is defined by $0=f\left(\mathbf{x}_{s}\right)$. In the alternative coordinates, the solution manifold is defined by $0=g\left(\mathbf{a}_{s}\right)$.

*Effect on Tolerance Cost*

To calculate tolerance cost, the observed data set is shifted to a new location in execution space without changing its distribution and the corresponding average result is evaluated. This is repeated for shifts to all points on a grid covering an experimentally reasonable range of execution space to find the shift that yields the minimum average result. The difference between the original average result and the minimum is tolerance cost.

Consider a particular observed execution (expressed in either coordinate frame) and the corresponding result, $r_{i}=f\left(\mathbf{x}_{i}\right)=f\left(\mathbf{t}\left(\mathbf{a}_{i}\right)\right)=g\left(\mathbf{a}_{i}\right)$, where the subscript *i* denotes any initial observation. To shift the data set without changing its distribution, the same constant vector, $\mathbf{x}_{c}$, is added to each data point, yielding a new result $r_{i,new}=f\left(\mathbf{x}_{i}+\mathbf{x}_{c}\right)=f\left(\mathbf{t}\left(\mathbf{a}_{i}\right)+\mathbf{t}\left(\mathbf{a}_{c}\right)\right)$. In the other coordinates, adding the corresponding constant vector, $\mathbf{a}_{c}$, yields an alternative new result, . In general it is different; however, the effect of adding a constant vector will be the same in both coordinates if $\mathbf{t}\left(\mathbf{a}_{i}+\mathbf{a}_{c}\right)=\mathbf{t}\left(\mathbf{a}_{i}\right)+\mathbf{t}\left(\mathbf{a}_{c}\right)$. That is one of the conditions for the coordinate transformation to be linear. If $\mathbf{x}=\mathbf{Ta}$, where **T** is a matrix of constants, $r_{i,new}=f\left(\mathbf{x}_{i}+\mathbf{x}_{c}\right)=f\left(\mathbf{Ta}_{i}+\mathbf{Ta}_{c}\right)=f\left(\mathbf{T}\left(\mathbf{a}_{i}+\mathbf{a}_{c}\right)\right)=g\left(\mathbf{a}_{i}+\mathbf{a}_{c}\right)=r_{i,new,alt}$. Thus tolerance cost is not affected by a linear transformation of coordinates.

*Effect on Noise Cost*

To calculate noise cost the observed data is shrunk towards its mean, without changing the shape of its distribution, in successive incremental steps until a minimum average result is obtained. The difference between the original average result and the minimum is noise cost.

Consider a linear transformation as above but now applied to the noise cost calculations. At each step the same amount $\Delta\mathbf{x}$ is subtracted from each initial data point $\mathbf{x}_{i}$, where $\Delta\mathbf{x}$ is a small fraction *s* (typically 1%) of the distance of $\mathbf{x}_{i}$ from the mean of the data distribution, $\mathbf{\mu}_{x}$. $\Delta\mathbf{x}=s\mathbf{I}\left(x_{i}-\mathbf{\mu}_{x}\right)$ where *s* = 0.01 and **I** is the unit matrix. The corresponding change of result $\Delta r$ may be approximated as $\Delta r\cong\nabla\mathbf{f}^{t}\Delta\mathbf{x}$ where $\nabla\mathbf{f}^{t}=\left[\partial f/\partial\mathbf{x}\right]$ is the *Jacobian* (or Jacobian matrix), a matrix of first partial derivatives of the result function with respect to its arguments. With two execution variables, $r=f\left(x_{1},x_{2}\right)$, $\nabla\mathbf{f}^{t}=\left[\begin{matrix} \partial f/\partial x_{1} & \partial f/\partial x_{2} \end{matrix}\right]$ and $\Delta r\cong\frac{\partial f}{\partial x_{1}}\Delta x_{1}+\frac{\partial f}{\partial x_{2}}\Delta x_{2}$. If the same operation is performed in alternative coordinates, the change of result (with a self-evident notation) becomes $\Delta r_{alt}\cong\nabla\mathbf{g}^{t}\Delta\mathbf{a}$. Using the chain rule for differentiation, the Jacobians in the two coordinates are related by $\nabla\mathbf{g}^{t}=\nabla\mathbf{f}^{t}\mathbf{T}$. The change of result in alternative coordinates may be expressed as $\Delta r_{alt}\cong\nabla\mathbf{f}^{t}\mathbf{T}\Delta\mathbf{a}$. Using $\Delta\mathbf{x}=\mathbf{T}\Delta\mathbf{a}$, the change of result in original coordinates may be expressed as $\Delta r\cong\nabla\mathbf{f}^{t}\mathbf{T}\Delta\mathbf{a}$. Thus $\Delta r_{alt}=\Delta r$ and the change of result is the same when the calculation is performed in either coordinate frame. Applying this calculation to all points in a data set it can be seen that (to within the accuracy of the approximation used above) a linear transformation of execution coordinates does not affect noise cost.

The accuracy of the approximation is determined by the curvature of the result function; that is, how well its curved surface may be approximated by a tangent plane in the region occupied by the data. Extending this argument to nonlinear coordinate transformations, if they, too, have sufficiently small curvature in the region occupied by experimental data, noise cost will be minimally affected.
